# Prediction of Compressive Strength of Fly Ash Based Concrete Using Individual and Ensemble Algorithm

**DOI:** 10.3390/ma14040794

**Published:** 2021-02-08

**Authors:** Ayaz Ahmad, Furqan Farooq, Pawel Niewiadomski, Krzysztof Ostrowski, Arslan Akbar, Fahid Aslam, Rayed Alyousef

**Affiliations:** 1 Department of Civil Engineering, Abbottabad Campus, COMSATS University Islamabad, Islamabad 22060, Pakistan; ayazahmad@cuiatd.edu.pk; 2 Faculty of Civil Engineering, Wroclaw University of Science and Technology, Wybrzeże Wyspiańskiego 27, 50-370 Wroclaw, Poland; pawel.niewiadomski@pwr.edu.pl; 3 Faculty of Civil Engineering, Cracow University of Technology, 24 Warszawska Str., 31-155 Cracow, Poland; krzysztof.ostrowski.1@pk.edu.pl; 4 Department of Architecture and Civil Engineering, City University of Hong Kong, Kowloon, Hong Kong; 5 Department of Civil Engineering, College of Engineering in Al-Kharj, Prince Sattam Bin Abdulaziz University, Al-Kharj 11942, Saudi Arabia; f.aslam@psau.edu.sa (F.A.); r.alyousef@psau.edu.sa (R.A.)

**Keywords:** concrete compressive strength, fly ash waste, ensemble modeling, decision tree, DT-bagging regression, cross-validation python

## Abstract

Machine learning techniques are widely used algorithms for predicting the mechanical properties of concrete. This study is based on the comparison of algorithms between individuals and ensemble approaches, such as bagging. Optimization for bagging is done by making 20 sub-models to depict the accurate one. Variables like cement content, fine and coarse aggregate, water, binder-to-water ratio, fly-ash, and superplasticizer are used for modeling. Model performance is evaluated by various statistical indicators like mean absolute error (MAE), mean square error (MSE), and root mean square error (RMSE). Individual algorithms show a moderate bias result. However, the ensemble model gives a better result with R^2^ = 0.911 compared to the decision tree (DT) and gene expression programming (GEP). K-fold cross-validation confirms the model’s accuracy and is done by R^2^, MAE, MSE, and RMSE. Statistical checks reveal that the decision tree with ensemble provides 25%, 121%, and 49% enhancement for errors like MAE, MSE, and RMSE between the target and outcome response.

## 1. Introduction

Carbon dioxide produced from the cement industry has a malignant adamant effect on environmental conditions [1]. Its utilization and excessive use in modern construction around the world produces greenhouse gases (GHG) [2]. Moreover, countless amounts of gases are emitted during the production of cement due to the burning of natural resources and fossil fuels [3]. Annually, 4 billion tons of Portland cement (PC) is being produced and approximately one ton of cement generates one ton of CO_2_ gas [4]. This huge amount of carbon dioxide is a serious threat to the environment. The report shows that a 1.6% increment (3.4% to 5%) of global CO_2_ gas discharge was observed from the year 2000 to 2006. The cement industry contributes 18% of industrial greenhouse gases (GHG to the environment. This is due to the direct process-related activity, energy-related combustion, and remaining use of electricity, which is termed as indirect energy [5]. To overcome the above-mentioned issue, a process of replacing the cement material with an alternative binder is of great research interest [6].

The supplementary cementation materials (SCMs) can be used for many purposes, especially in the concrete industry. Their utilization in concrete gives a benignant effect by reducing the percentage of CO_2_ gas emitted. SCMs used in the cement industry can be industrial and agricultural waste products, which includes olive oil, bagasse ash, sugarcane, rice husk ash, palm oil fuel ash, etc. However, commonly adopted and used in the construction industry are silica fume, fly ash, and ground granulated blast furnace slag [7,8,9]. Their utilization in concrete reduces the malignant effect on the environment [10]. The replacement of cement in concrete with the waste material helps both in the utilization of the wastes and fulfills the increasing demand for the concrete. What is more, it has been observed that the use of waste materials as pozzolanic in high-strength concrete improves its strength and durability. This alternately helps minimize the impending environmental degradation [11].

Concrete is stated as the second-highest used material after water in the world [12]. This is due to the intensive use of concrete in the construction industries and the field of civil engineering. Concrete requires a comprehensive technique to produce. It is a mixture of multiple materials like coarse aggregate, fine aggregate, water, binder, admixtures, and supplementary raw materials [13]. The concrete matrix is the random distribution of the previously mentioned variables [14]. The extensive use of it can be seen as a building material around the globe. For the effective evaluation of the performance of concrete according to the advanced design technologies, its mechanical properties must be examined [14]. One of its supreme mechanical properties is its compressive strength, which is alternately the sign of structural safety throughout life [15]. This remarkable property of concrete can be affected by numerous factors, like particle size, water-to-cement ratio, waste composition, and use of chemicals. However, casting concrete by using the proper techniques in the laboratory and conducting experimental tests to find the mechanical properties after the setting is quite a time-consuming task [14]. Moreover, using the previously mentioned technique in the recent and modern period of life is quite uneconomical. Thus, the modern methodologies of machine learning techniques can be adapted to predict the desired result in advance [16]. The prediction of variables can be done from regressions and machine learning models. These algorithm-based techniques give a precise relation and predict the accurate model by the use of input variables [17].

Machine learning approaches are raising trends in the domain of civil engineering. They are extensively used in forecasting the mechanical properties of concrete [18,19,20,21]. These techniques use extensive data to build a precise model. Their prediction accuracy is dependent upon the data sample used in experimental work during casting of the specimen or upon the literature study. Researchers use these algorithms for the prediction of the mechanical properties of concrete. Javed et al. [22] predict the compressive strength of sugarcane bagasse ash (SCBA) concrete using gene expression programming (GEP). The author used the experimental test for calibration and validation of the model. Similarly, Aslam et al. [23] predict the compressive strength of high-strength concrete (HSC) by employing GEP. The author used 357 data points and reported an adamant relationship between the target and predicted values. Hosein et al. [24] forecast the mechanical properties of recycled concrete (REC) by using an artificial neural network (ANN). Correspondingly, Getahun et al. [25] forecast the strength of concrete incorporating waste materials using ANN. The author concluded that ANN gives adamant relation with fewer errors. Similarly, Qing et al. [26] predict the diffusion capability of chloride in reinforced concrete structures with ANN. The result indicates better prediction by employing an individual algorithm based on 653 data samples. Farooq et al. [15] predict the compressive nature of HSC by developing two models with random forest (RF) and GEP. RF gives a robust performance with precise correlation with strong predicted values. That machine learning algorithm is not limited to predict only the compressive or tensile nature of concrete but can be used to forecast any response in any engineering or data sciences domain. In turn, Ahmad et al. [27] employ supervised machine learning (ML) algorithms to predict energy in the distinct buildings. Similarly, Wang et al. [28] predict the COVID-19 response by employing different ML-based algorithms. Cladera et al. [29] predict the response of a structural beam with and without stirrups by using ANN. The author achieved a better response from modeled than empirical relations. Similarly, Onyari et al. [30] reveal robust performance by utilizing ANN to predict the flexural and compressive strength of modified mortar. Previously mentioned examples show the overwhelming response of individual algorithms.

Recently, application of ensemble modeling is perceived as a chance for enhancement of the model’s overall efficiency. It can be achieved due to taking a weak leaner to build strong, predictive learners than individual learners [31]. Feng et al. [32] use ensemble algorithm techniques for the prediction of failure mode classification and bearing capacity of reinforced concrete (RC) structural element (column). Both models give robust performance. However, bearing capacity is characterized by better correlation than failure mode classification. Bui et al. [33] employed a modified firefly algorithm with ANN on high performance concrete (HPC) and reported better performance of the model. Moreover, Salamai et al. [34] report good accuracy of R^2^ = 0.9867 by using the RF algorithm. In turn, Cai et al. [35] use various supervised machine ensemble algorithms for the prediction of chloride penetration in the RC structure situated in a marine environment. Ensemble models outclass individual algorithms to predict chloride penetration in RC. Hacer et al. [36] present the comparative assessment of bagging as the ensemble approach for high-performance concrete mix slump flow. Ensemble models with bagging were found to be superior with regard to standalone approaches. Halil et al. [37] predict the strength of HPC by employing three ensemble modeling approaches. The author used the decision tree as a base learner for other models and found that the hybrid model outperforms with the output result of R^2^ = 0.9368 among the several proposed models. Kermani et al. [38] represents the performance of five soft, computing base learners for predicting concrete corrosion in sewers. The author used both tree-based and network-based learners and reported that RF ensemble learners give a better result with R^2^ = 0.872. These ensemble approaches give an enhanced effect with robust performance of the overall models.

**Figure 1:**
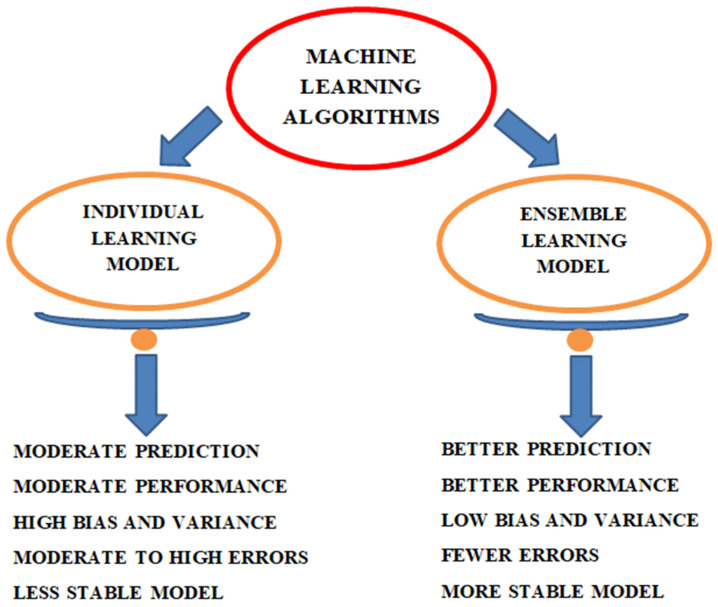
Comparison between individual and ensemble approaches.

Taking the above into consideration, it may seem that ensemble learning models have more favorable features and give better results than individual learning models. The difference between individual and ensemble model is illustrated in Figure 1.

## 2. Research Significance

The aim of this study is to use the machine-learning algorithm with ensemble modeling using Anaconda Python to predict the compressive strength of fly-ash-based concrete using different algorithms. A decision tree with a bagging algorithm is used and optimization is done by making 20 sub-models to give a strong outcome. A comparison is made with the individual, ensemble algorithms, and with gene expression programming to give the best model. Moreover, K-fold cross-validation and a statistical check are applied to evaluate the model performance.

## 3. Data Description

**Figure 2:**
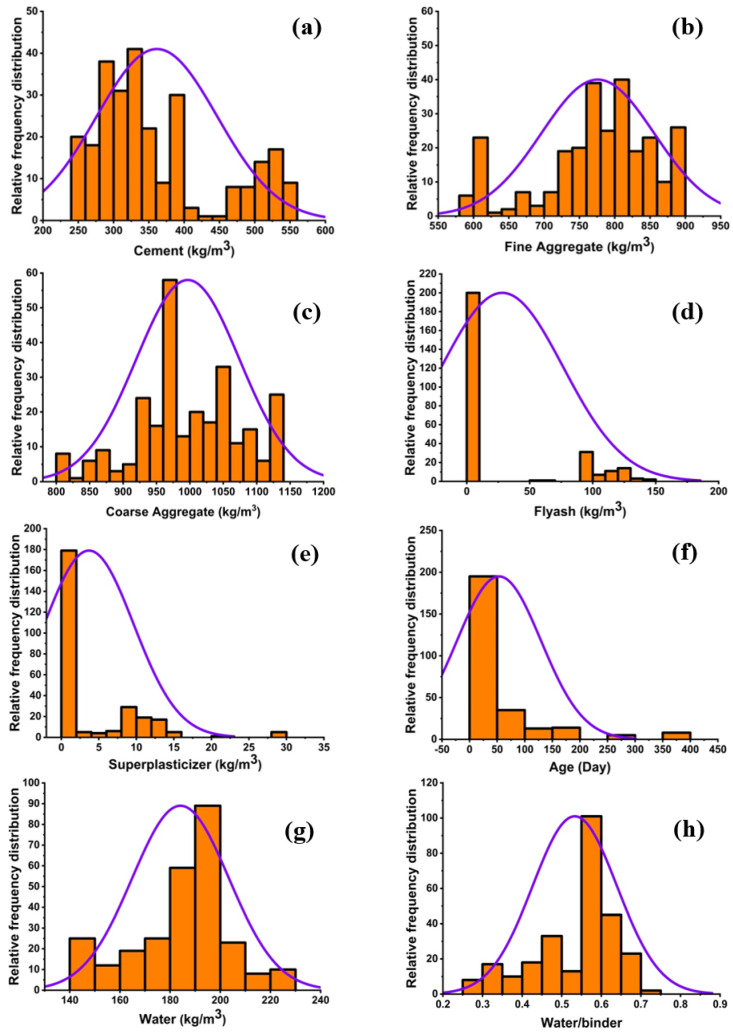
Relative frequency distribution of variables, (**a**) cement, (**b**) fine aggregate, (**c**) coarse aggregate, (**d**) fly ash, (**e**) superplasticizer, (**f**) age, (**g**) water, and (**h**) water-to-binder ratio.

**Figure 3:**
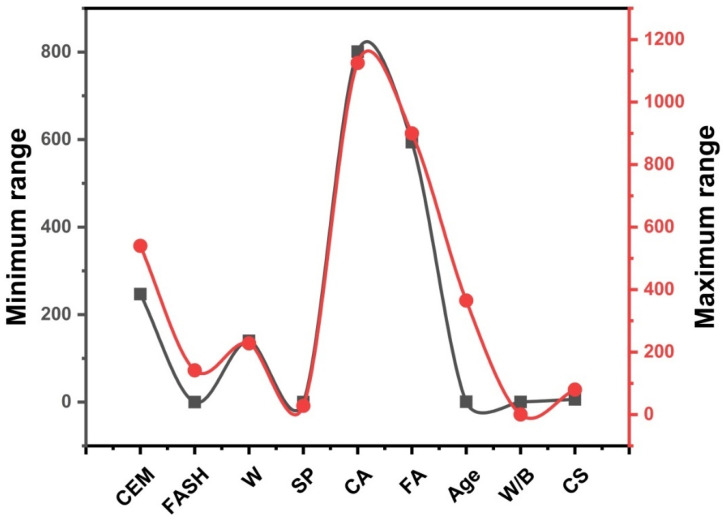
Parameters range of variables with minimum and maximum values.

The efficiency of the model is completely dependent upon the variables and the number of data samples used. The parameters used in models preparation in order to predict the strength of concrete were taken from published literature [39] and are summarized in Appendix A. Eight variables concerning composition of the concrete mixture and including cement, fine and coarse aggregate, superplasticizer, water, waste material, age, and a water-to-binder ratio were taken into analysis. The overall distribution in terms of the relative frequency distribution is illustrated in Figure 2. The range of variables of each parameter used in the study, with a minimum and maximum value, is illustrated in Figure 3. Statistical descriptive analysis for the variables in terms of strength is listed in Table 1.

## 4. Methodology

**Figure 4:**
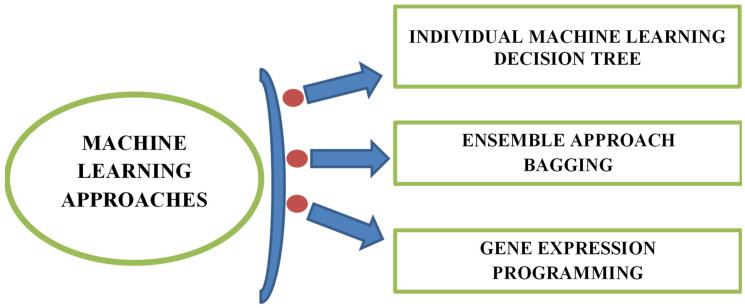
Machine learning model methodology.

Individual and ensemble model techniques used to predict the properties in a limited time that are of great interest. The accuracy level between the actual and prediction level is typically obtained from the R^2^ value (ranges from 0–0.99). A high R^2^ value indicates the satisfactory results of the selected technique. This study uses three approaches to predict the compressive strength of concrete with waste material. A decision tree with ensemble algorithms such as bagging with a learning rate of 0.9 and gene expression programming is used. These techniques are selected due to their popularity among other algorithms. The overall machine learning model methodology in the form of a diagram is illustrated in Figure 4.

### 4.1. Decision Tree

The decision tree is one of the supervised learning techniques used for categorizing regression problems but is also commonly used for classification problems [40]. There are classes inside the tree. However, if there is no class, then the regression technique can predict the outcome by independent variables [37]. A decision tree is a tree-structured classifier in which the inner nodes reflect the attribute of a database. Branches indicate the conclusion rules, and every leaf node constitutes the outcome. The decision tree consists of two nodes known as a decision node and a leaf node. Decision nodes have multiple branches with the capability to make any decision, while leaf nodes do not have branches and are considered as the output of the decisions. It is known as a decision tree because it has a similar nature to a tree that starts with the root node and distributes in the number of branches, and reflects a tree-like structure [41]. The decision tree splits the data samples at various points. The executed algorithm finds the error between the target and predicted value at every divided point. The errors are calculated at every divided point, and the variable with the least value for the fitness function is selected as a split point, and the same procedure is repeated again.

### 4.2. Ensemble Bagging Approach

The ensemble technique is the concept of machine learning used to train numerous models by applying a similar learning algorithm [42]. The ensemble involves a substantial group of methods known as multi-classifiers. The group of hundreds or thousands of learners with a common intent are joined together to fix the problem. Bagging is a parallel type ensemble method that explains the variance of the prediction model by producing supplementary data in the stage of training. This production is from irregular sampling including substituting from the real set of data. Some of the observations can be repeated by sampling with replacement in every new training data set. In bagging, every component has an equal chance to appear in the new dataset. The force of prediction cannot be enhanced by increasing the size of the training set. The variance can also be reduced narrowly by tuning the forecast to an anticipated outcome. All these numbers of sets of the given data are normally used to train other numbers of models. This ensemble of different models uses the average of all the predictions from the other various models. In regression, the prediction may be the mean or average of the predictions taken from the different models [43]. The decision tree with bagging is tuned with 20 sub-models to obtain the optimized value that gives an adamant output result.

### 4.3. Gene Expression Programming

Gene expression programming (GEP) is a computer programming-based algorithm used to develop different models [44]. GEP, which is initially introduced by Ferreira [45], is considered to be a natural development of genetic programming (GP). Multiple numbers of genetic operators that are being used in genetic algorithms (GAs) can also be used in GEP with the help of a few recommended changes. There are five main components of GEP, namely, function set, terminal set, fitness function, control variables, and termination condition. GEP works as a fixed length of character twine to explain the problems, which are next defined as tree-like structures with different dimensions. This type of tree is known as the GEP expression tree (ETs). Selection of individual chromosomes takes place and then they are copied into the next generation, as per the fitness by roulette wheel sampling with elitism [23]. This ensures the durability and replication of the best individual to the next generation. Fluctuation in the population is shown by applying one or more genetic operators (mutation, crossover, or rotation) on the given chromosomes. Among the number of advantages of GEP, the formation of genetic diversity is remarkably simplified because of the working of genetic operators at the chromosome level. This multi-genic approach of GEP permits the natural selection of other complicated and complex programs composed of numerous subprograms. GEP genes along with a function set and terminal set play a vital role during the process [46].

### 4.4. K-Fold Cross-Validation and Statistical Measures

**Figure 5:**
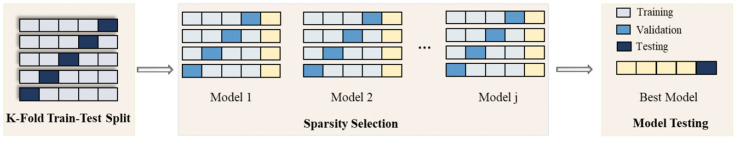
K-fold cross-validation algorithm [46].

The model performance in terms of bias and variance is checked by employing K-fold cross-validation. The data is divided into 10 stratified groups, which randomly distribute the data into a training set and test set. This process takes one part of the overall data into the test sample and the remaining into the training set, as illustrated in Figure 5. The model’s overall efficiency by cross-validation is then tested by taking an average of 10 rounds by various errors. Similarly, the model evaluation is also done by using statistical indicators [23]. Three types of the indicator are used in our current study, which is listed below (Equations (1)–(3)).

(1) $$\mathrm{MAE}\;=\;\frac{1}{n}\overset{n}{\underset{i\;=\;1}{\sum }}\left|x_{i}-x\right|$$

(2) $$\mathrm{MSE}\;=\;\frac{1}{n}\overset{n}{\underset{i\;=\;1}{\sum }}{\left(y_{pred}-y_{ref}\right)}^{2}$$

(3) $$\mathrm{RMSE}\;=\;\sqrt{\sum \frac{{\left(y_{pred}-y_{ref}\right)}^{2}}{n}}$$

where:

- *n* = Total number of data samples,
- $x,y_{ref}$ = reference values in the data sample,
- $x_{i},y_{pred}$ = predicted values from models.

## 5. Model Result

### 5.1. Decision Tree/Ensemble Model

**Figure 6:**
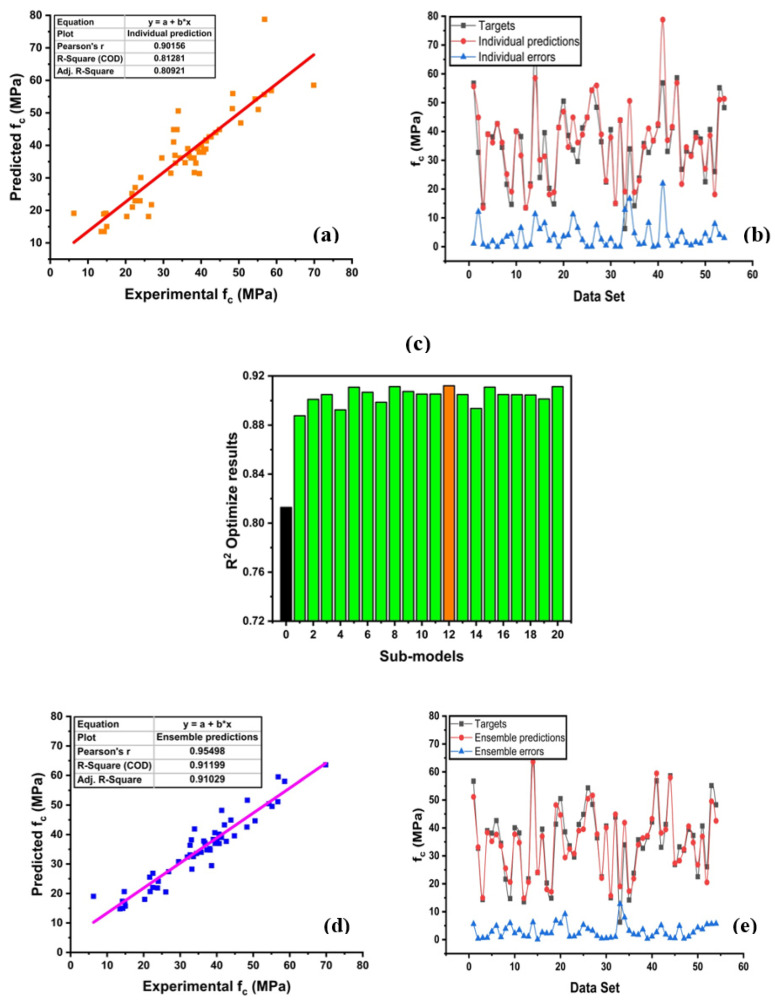
Decision tree (DT) with the ensemble model. (**a**) Predicted regression model with DT. (**b**) Model errors between targets and predictions from the DT technique. (**c**) Optimize model of ensemble. (**d**) Predicted regression model with DT-bagging. (**e**) Model Errors between targets and predictions from the DT-bagging technique.

The prediction of concrete strength by employing a decision tree yields an adamantly strong relationship between targets to output strength, as depicted in Figure 6. It can be seen that the individual model gives a better response with less variance, as illustrated in Figure 6a. However, the decision tree with bagging gives precise performance than an individual one, as illustrated in Figure 6d. This is due to an increase in model efficiency as it takes several data to train the best model by using weak base learners [47]. The ensemble model is optimized by making 20 sub-models, as depicted in Figure 6c. The zero number shows the individual model, which is made by using the decision approach and shows R^2^ = 0.812. After the ensemble approach, there is a significant enhancement in the overall response of the model. Every model shows a surpass effect by giving an average score of about R^2^ = 0.904 within 20 models. However, the 12th sub-model gives a prime result with R^2^ = 0.911, as depicted in Figure 6c. Moreover, the model comparison in terms of errors is depicted in Figure 6b,e. Decision tree (DT) with bagging enhances the model accuracy by giving fewer errors. The test data shows that there is a 20.10% prediction capacity of average errors by bagging than in the individual model. Besides, DT shows the minimum and maximum error of 0 and 21.97 MPa, respectively. Similarly, DT with an ensemble model shows the minimum and maximum error of 0.11, and 12.77 MPa, respectively. The detailed result is shown in Table 2.

### 5.2. Gene Expression Programming

**Figure 7:**
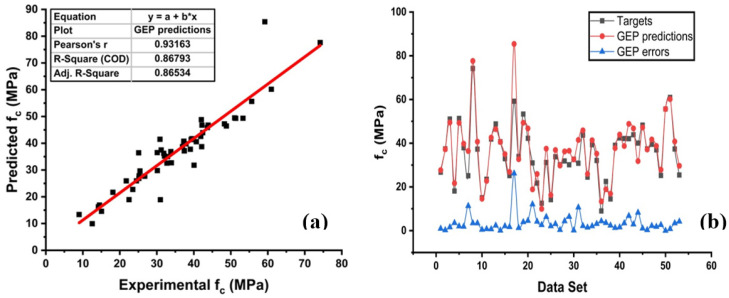
Gene expression programming (GEP) model: (**a**) Predicted regression model. (**b**) Model Errors between targets and predictions from the GEP model.

The performance of the model by GEP yielded a robust relationship between targets and predicted, as illustrated in Figure 7. It can be seen that R^2^ by employing GEP is close to 1. Moreover, Figure 7b represents the error distribution of the testing set with fewer errors. Similarly, the predicted value shows a lower error to target values with a minimum, maximum, and average value of 0.00 MPa, 26.20 MPa, and 3.48 MPa, respectively. Table 2 presents detailed results from the models.

### 5.3. Evaluation of the Model by K-Fold and Statistical Checks

Cross-validation is a statistical practice used to evaluate or estimate the actual performance of the machine learning models. It is necessary to know the performance of the selected models. For this purpose, a validation technique is required to find the accuracy level of the model’s data. Shuffling of the data set randomly and splitting a dataset into k-groups is required for the k-fold validation test. In the described study, data of experimental samples are equally divided into 10 subsets. It uses nine out of ten subsets, while the only subset is utilized for the validation of the model. The same approach of this process is then repeated 10 times for obtaining the average accuracy of these 10 repetitions. It is clarified widely that the 10-fold cross-validation method well represents the conclusion and accuracy of the model performance [48].

**Figure 8:**
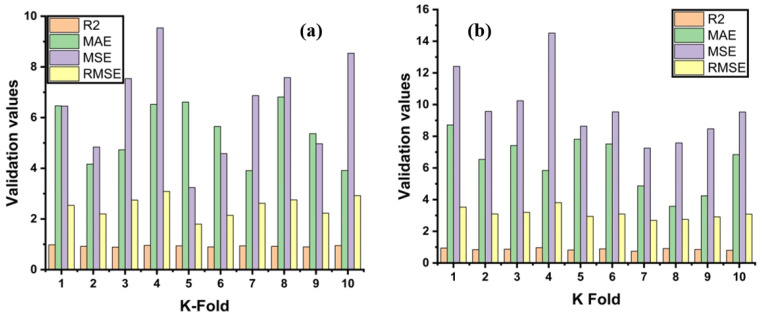
Statistical indicators from K-Fold Cross-validation; (**a**) Ensemble model; (**b**) GEP model.

Bias and a variance decrease for the test set can be checked by employing K-fold cross-validation. The results of cross-validation are evaluated by a correlation coefficient (R^2^), a mean absolute error (MAE), a mean square error (MSE), and a root mean square error (RMSE), as illustrated in Figure 8. The ensemble model shows fewer errors and better R^2^ as compared to GEP. The average R^2^ for ensemble modeling is 0.905 with a maximum and minimum values of 0.84 and 0.96, as depicted in Figure 8a. Whereas the GEP model shows an average R^2^ = 0.873 of ten folds with 0.76 and 0.95 for a minimum and maximum correlation, respectively, as shown in Figure 8b. Each model shows fewer errors for validation. The validation indicator result shows that ensemble means values of MAE, MSE, and RMSE come to be 6.43 MPa, 6.66 MPa, and 2.55 MPa, respectively. Similarly, the GEP model shows the same trend by showing fewer errors. The GEP model shows mean values of 7.30 MPa, 9.60 MPa, and 3.06 MPa for MAE, MSE, and RMSE, respectively (see Figure 8b). Table 3 represents the validation results of both models.

Statistical check is also applied to evaluate the model with regard to the testing results. The statistical check is an indicator that shows the model response towards prediction, as shown in Table 4. It can be seen that models depict bottom-most errors. However, the ensemble model shows a 25% error reduction for MAE as compared to the individual and GEP. Similarly, the bagging approach indicates the robust performance of the model. Moreover, MSE and RMSE for strong learners show 121% and 49% enhancement in the predictions by showing reduced errors between the target and predicted outcomes, as shown in Table 4.

**Figure 9:**
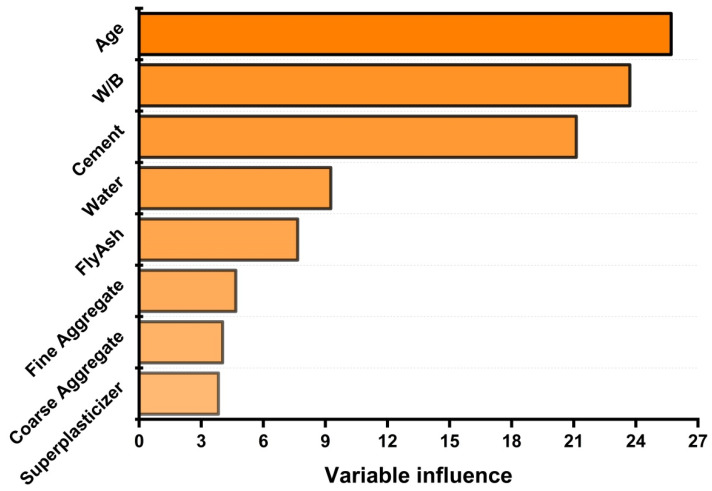
Variable influence on compressive strength of fly ash-based concrete.

Moreover, permutation feature importance via python is conducted to check the influence of variables on strength, as depicted in Figure 9. These variables have a vital influence on the prediction of compressive strength of concrete. The concrete age, cement, and water-to-cement ratio have a significant influence on model analysis. Whereas water, filler material (fly ash), superplasticizer, fine aggregate, and coarse aggregate have moderate influences in making the model. Thus, it can be concluded that every parameter is crucial in the forecasting of the strength properties. However, cement, age, and the water-to-cement ratio should be given more importance while casting of specimens.

### 5.4. Limitation and Future Work

Despite the fact that, in the work, a thorough analysis based on a large number of data points was conducted and an extensive machine learning algorithm with evaluation was implemented, the limitations of work should be mentioned. Described in the paper selection, an approach can be enhanced by using other appropriate methods. A clear limitation of work is the number of data points equal to 270. The study is also limited to predict only one result from various mechanical properties of concrete. Tensile strength, durability, corrosion, toughness, and abrasion behavior of concrete is not considered in this work. Other algorithm-based techniques, like artificial neural network (ANN), support vector machine (SVM), gradient boosting, and AdaBoost may also be applied to the same dataset for a better understanding. However, this research work does not only focus on algorithm-based techniques but also involves the programming-based GEP, which indicated the wide scope of this work.

Since concrete is the most widely used material after water on this earth, it is further recommended that other properties of this material should be incorporated except for its compressive strength. Machine learning techniques should also be used to predict the environmental effects on concrete properties. To achieve high accuracy in the actual and predicted results, the multi-stage genetic programming approach may also be used. It is also recommended that models can be run for the concrete modified with different fibers as: jute fibers, glass fibers, polypropylene fibers, nylon fibers, and steel fibers.

## 6. Conclusions

This study describes the supervised machine learning approaches with ensemble modeling and gene expression programming to predict concrete strength. The following points are drawn from the analysis:

1. A decision tree with ensemble modeling gives a robust performance compared to a decision tree individually and with gene expression programming. The correlation coefficient of R^2^ = 0.911 is reported for DT with bagging.
2. Optimization of the model for the decision tree with bagging is done by making twenty sub-models. Magnificent enhancement is observed from the twelve, which shows R^2^ = 0.911 as compared to the individual model with R^2^ = 0.812.
3. Validation score is conducted by different indicators. Both models (DT with bagging and GEP) show better anticipation for testing results.
4. Statistical analysis checks reveal that the decision tree with bagging shows enhancement in model accuracy by minimizing the error difference between targeted and predicted values.

To summarize, all applied algorithms show a significant effect on the model’s quality by predicting the target response more accurately. As described in the paper, machine learning approaches can save experimental time and predict the outcome by gathering extensive data from laboratory and published papers. It can help the scientific society to predict the properties and responses in the coming month or year.

## Figures and Tables

**Table 1:** Statistical measures on variables.

Statistics	Cem *	FASH *	W *	SP *	CA *	FA *	Age *	W/B *
Mean	361.39	28.15	184.15	3.68	996.90	775.93	53.31	0.53
Standard Error	5.20	2.94	1.17	0.36	4.70	4.86	4.63	0.01
Median	336.25	0.00	189.00	0.00	987.80	781.95	28.00	0.58
Mode	349.00	0.00	192.00	0.00	1125.00	613.00	28.00	0.58
Standard Deviation	85.49	48.35	19.29	5.95	77.26	79.92	76.06	0.11
Sample Variance	7309.14	2337.79	372.16	35.39	5969.32	6387.59	5784.50	0.01
Kurtosis	−0.50	−0.44	0.29	3.52	−0.19	−0.07	7.01	−0.04
Skewness	0.83	1.20	−0.38	1.77	−0.26	−0.67	2.62	−0.92
Range	293.20	142.00	88.00	28.20	324.00	305.80	364.00	0.43
Minimum	246.80	0.00	140.00	0.00	801.00	594.00	1.00	0.27
Maximum	540.00	142.00	228.00	28.20	1125.00	899.80	365.00	0.70
Sum	97,574.60	7601.70	49,720.30	993.40	269,163.90	209,502.40	14,394.00	143.89
Count	270.00	270.00	270.00	270.00	270.00	270.00	270.00	270.00

* CEM = Cement (kg/m^3^), FASH = Fly ash (kg/m^3^), W = Water (kg/m^3^), SP = Super plasticizer (kg/m^3^), CA = Coarse aggregate (kg/m^3^), FA = Fine aggregate (kg/m^3^), and W/B = Water to binder.

**Table 2:** Evaluation of models.

Data Points	Decision Tree (DT) Targets	DT Predictions	Ensemble Prediction	Gene Expression Programming (GEP) Targets	GEP Predictions	DT Errors	Ensemble Errors	GEP Errors
1	56.74	55.64	51.14	26.74	27.66	1.10	5.60	0.92
2	32.72	44.87	33.11	37.44	37.21	12.15	0.39	0.23
3	14.31	13.52	14.94	51.04	49.48	0.79	0.63	1.56
4	39.06	39.05	38.35	18.13	21.68	0.01	0.71	3.55
5	38.11	36.15	35.22	51.33	49.31	1.96	2.89	2.02
6	42.64	42.64	37.67	37.91	39.76	0.00	4.97	1.85
7	34.49	36.15	33.57	25.10	36.42	1.66	0.92	11.32
8	21.65	25.18	25.55	74.17	77.61	3.53	3.90	3.44
9	14.7	19.11	20.62	37.27	40.69	4.41	5.92	3.42
10	40.06	40.06	37.74	15.05	14.58	0.00	2.32	0.47
11	38.21	31.65	34.74	23.52	22.76	6.56	3.47	0.76
12	13.52	13.52	14.79	41.89	42.56	0.00	1.27	0.67
13	21.78	21.02	20.63	48.79	46.44	0.76	1.15	2.35
14	69.84	58.52	63.61	40.68	40.59	11.32	6.23	0.09
15	24	30.14	24.11	32.92	34.99	6.14	0.11	2.07
16	39.58	31.35	37.01	25.18	26.87	8.23	2.57	1.69
17	20.28	18.13	18.00	59.20	85.40	2.15	2.28	26.20
18	14.84	18.91	17.15	33.94	32.67	4.07	2.31	1.27
19	41.37	41.37	48.22	53.30	49.35	0.00	6.85	3.95
20	50.51	46.9	44.65	42.22	46.77	3.61	5.86	4.55
21	38.6	34.57	29.43	30.96	18.90	4.03	9.17	12.06
22	33.61	44.87	32.51	21.75	25.93	11.26	1.10	4.18
23	29.59	36.15	30.80	12.54	9.95	6.56	1.21	2.59
24	41.24	38.89	39.02	31.18	37.50	2.35	2.22	6.32
25	44.86	44.87	39.55	14.20	16.26	0.01	5.31	2.06
26	54.32	54.28	50.46	33.80	36.88	0.04	3.86	3.08
27	48.4	55.94	51.65	30.14	29.77	7.54	3.25	0.37
28	36.45	39	37.80	31.88	36.23	2.55	1.35	4.35
29	22.5	22.95	22.03	30.12	36.52	0.45	0.47	6.40
30	40.66	37.91	40.12	32.72	32.57	2.75	0.54	0.15
31	14.99	15.05	15.71	30.85	41.47	0.06	0.72	10.62
32	43.89	43.94	44.89	43.70	45.88	0.05	1.00	2.18
33	6.27	19.11	19.05	24.50	25.95	12.84	12.78	1.45
34	33.94	50.6	41.89	39.29	41.35	16.66	7.95	2.06
35	14.2	18.91	17.38	32.07	35.21	4.71	3.18	3.14
36	23.8	22.95	21.86	9.01	13.37	0.85	1.94	4.36
37	35.76	34.68	33.99	22.50	18.93	1.08	1.77	3.57
38	32.72	41.05	36.38	14.50	16.89	8.33	3.66	2.39
39	36.8	36.8	37.17	39.06	37.75	0.00	0.37	1.31
40	42.13	42.62	43.28	42.42	43.95	0.49	1.15	1.53
41	56.83	78.8	59.50	42.13	38.72	21.97	2.67	3.41
42	33.08	36.94	38.20	42.03	48.82	3.86	5.12	6.79
43	41.3	41.64	39.45	43.89	46.77	0.34	1.85	2.88
44	58.61	56.85	58.01	40.06	31.79	1.76	0.60	8.27
45	26.85	21.75	27.39	48.28	47.22	5.10	0.54	1.06
46	33.21	34.57	28.27	37.42	37.11	1.36	4.94	0.31
47	31.97	31.45	32.39	39.49	41.69	0.52	0.42	2.20
48	39.49	37.91	40.61	36.94	38.73	1.58	1.12	1.79
49	37.33	36.15	34.77	25.22	27.85	1.18	2.56	2.63
50	22.53	27.04	26.86	55.64	55.64	4.51	4.33	0.00
51	40.68	38.63	36.96	60.95	60.16	2.05	3.72	0.79
52	26.06	18.13	20.51	37.33	40.76	7.93	5.55	3.43
53	55.16	51.04	49.54	25.45	29.65	4.12	5.62	4.20
54	48.28	51.33	42.55	-	-	3.05	5.73	-

**Table 3:** Result of K-Fold Cross-validation.

K Fold	Ensemble Model				GEP Model			
	R^2^	MAE	MSE	RMSE	R^2^	MAE	MSE	RMSE
1	0.96	8.46	4.45	2.10	0.86	10.71	13.57	3.68
2	0.91	5.17	7.44	2.72	0.94	7.45	7.97	2.82
3	0.84	3.73	8.54	2.92	0.89	6.18	11.24	3.35
4	0.90	9.52	5.84	2.41	0.95	5.84	14.51	3.80
5	0.94	6.81	6.44	2.53	0.93	7.81	9.64	3.10
6	0.90	5.65	5.88	2.42	0.86	7.51	6.51	2.55
7	0.85	7.91	6.87	2.62	0.81	8.47	7.25	2.69
8	0.88	5.81	9.85	3.13	0.76	6.58	7.58	2.75
9	0.95	6.37	4.97	2.22	0.84	5.64	9.47	3.07
10	0.92	4.92	6.35	2.51	0.89	6.84	8.35	2.88

**Table 4:** Statistical checks.

Statistics	MAE (MPa)	MSE (MPa)	RMSE (MPa)
DT	3.896	36.01	6.00
DT-BAG	3.113	16.28	4.03
GEP	3.478	29.91	5.46

## Data Availability

The data presented in this article is available within the article.

## Appendix A

**Table A1:** Experimental variable data.

S. No.	Cement	Fly Ash	Water	Super Plasticizer	Coarse Aggregate	Fine Aggregate	Days	W/C	Strength
1	540	0	162	2.5	1040	676	28	0.3	79.99
2	540	0	162	2.5	1055	676	28	0.3	61.89
3	475	0	228	0	932	594	28	0.48	39.29
4	380	0	228	0	932	670	90	0.6	52.91
5	475	0	228	0	932	594	180	0.48	42.62
6	380	0	228	0	932	670	365	0.6	52.52
7	380	0	228	0	932	670	270	0.6	53.3
8	475	0	228	0	932	594	7	0.48	38.6
9	475	0	228	0	932	594	270	0.48	42.13
10	475	0	228	0	932	594	90	0.48	42.23
11	380	0	228	0	932	670	180	0.6	53.1
12	349	0	192	0	1047	806.9	3	0.55	15.05
13	475	0	228	0	932	594	365	0.48	41.93
14	310	0	192	0	971	850.6	3	0.62	9.87
15	485	0	146	0	1120	800	28	0.3	71.99
16	531.3	0	141.8	28.2	852.1	893.7	3	0.27	41.3
17	531.3	0	141.8	28.2	852.1	893.7	7	0.27	46.9
18	531.3	0	141.8	28.2	852.1	893.7	28	0.27	56.4
19	531.3	0	141.8	28.2	852.1	893.7	56	0.27	58.8
20	531.3	0	141.8	28.2	852.1	893.7	91	0.27	59.2
21	290.4	96.2	168.1	9.4	961.2	865	3	0.58	22.5
22	290.4	96.2	168.1	9.4	961.2	865	14	0.58	34.67
23	290.4	96.2	168.1	9.4	961.2	865	28	0.58	34.74
24	290.4	96.2	168.1	9.4	961.2	865	56	0.58	45.08
25	290.4	96.2	168.1	9.4	961.2	865	100	0.58	48.97
26	277.1	97.4	160.6	11.8	973.9	875.6	3	0.58	23.14
27	277.1	97.4	160.6	11.8	973.9	875.6	14	0.58	41.89
28	277.1	97.4	160.6	11.8	973.9	875.6	28	0.58	48.28
29	277.1	97.4	160.6	11.8	973.9	875.6	56	0.58	51.04
30	277.1	97.4	160.6	11.8	973.9	875.6	100	0.58	55.64
31	295.7	95.6	171.5	8.9	955.1	859.2	3	0.58	22.95
32	295.7	95.6	171.5	8.9	955.1	859.2	14	0.58	35.23
33	295.7	95.6	171.5	8.9	955.1	859.2	28	0.58	39.94
34	295.7	95.6	171.5	8.9	955.1	859.2	56	0.58	48.72
35	295.7	95.6	171.5	8.9	955.1	859.2	100	0.58	52.04
36	251.8	99.9	146.1	12.4	1006	899.8	3	0.58	21.02
37	251.8	99.9	146.1	12.4	1006	899.8	14	0.58	33.36
38	251.8	99.9	146.1	12.4	1006	899.8	28	0.58	33.94
39	251.8	99.9	146.1	12.4	1006	899.8	56	0.58	44.14
40	251.8	99.9	146.1	12.4	1006	899.8	100	0.58	45.37
41	249.1	98.8	158.1	12.8	987.8	889	3	0.63	15.36
42	249.1	98.8	158.1	12.8	987.8	889	14	0.63	28.68
43	249.1	98.8	158.1	12.8	987.8	889	28	0.63	30.85
44	249.1	98.8	158.1	12.8	987.8	889	56	0.63	42.03
45	249.1	98.8	158.1	12.8	987.8	889	100	0.63	51.06
46	252.3	98.8	146.3	14.2	987.8	889	3	0.58	21.78
47	252.3	98.8	146.3	14.2	987.8	889	14	0.58	42.29
48	252.3	98.8	146.3	14.2	987.8	889	28	0.58	50.6
49	252.3	98.8	146.3	14.2	987.8	889	56	0.58	55.83
50	252.3	98.8	146.3	14.2	987.8	889	100	0.58	60.95
51	246.8	125.1	143.3	12	1086.8	800.9	3	0.58	23.52
52	246.8	125.1	143.3	12	1086.8	800.9	14	0.58	42.22
53	246.8	125.1	143.3	12	1086.8	800.9	28	0.58	52.5
54	246.8	125.1	143.3	12	1086.8	800.9	56	0.58	60.32
55	246.8	125.1	143.3	12	1086.8	800.9	100	0.58	66.42
56	275.1	121.4	159.5	9.9	1053.6	777.5	3	0.58	23.8
57	275.1	121.4	159.5	9.9	1053.6	777.5	14	0.58	38.77
58	275.1	121.4	159.5	9.9	1053.6	777.5	28	0.58	51.33
59	275.1	121.4	159.5	9.9	1053.6	777.5	56	0.58	56.85
60	275.1	121.4	159.5	9.9	1053.6	777.5	100	0.58	58.61
61	297.2	117.5	174.8	9.5	1022.8	753.5	3	0.59	21.91
62	297.2	117.5	174.8	9.5	1022.8	753.5	14	0.59	36.99
63	297.2	117.5	174.8	9.5	1022.8	753.5	28	0.59	47.4
64	297.2	117.5	174.8	9.5	1022.8	753.5	56	0.59	51.96
65	297.2	117.5	174.8	9.5	1022.8	753.5	100	0.59	56.74
66	376	0	214.6	0	1003.5	762.4	3	0.57	16.28
67	376	0	214.6	0	1003.5	762.4	14	0.57	25.62
68	376	0	214.6	0	1003.5	762.4	28	0.57	31.97
69	376	0	214.6	0	1003.5	762.4	56	0.57	36.3
70	376	0	214.6	0	1003.5	762.4	100	0.57	43.06
71	500	0	140	4	966	853	28	0.28	67.57
72	475	59	142	1.9	1098	641	28	0.3	57.23
73	505	60	195	0	1030	630	28	0.39	64.02
74	451	0	165	11.3	1030	745	28	0.37	78.8
75	516	0	162	8.2	801	802	28	0.31	41.37
76	520	0	170	5.2	855	855	28	0.33	60.28
77	528	0	185	6.9	920	720	28	0.35	56.83
78	520	0	175	5.2	870	805	28	0.34	51.02
79	385	136	158	20	903	768	28	0.41	55.55
80	500.1	0	200	3	1124.4	613.2	28	0.4	44.13
81	405	0	175	0	1120	695	28	0.43	52.3
82	516	0	162	8.3	801	802	28	0.31	41.37
83	475	0	162	9.5	1044	662	28	0.34	58.52
84	500	0	151	9	1033	655	28	0.3	69.84
85	436	0	218	0	838.4	719.7	28	0.5	23.85
86	289	0	192	0	913.2	895.3	90	0.66	32.07
87	289	0	192	0	913.2	895.3	3	0.66	11.65
88	393	0	192	0	940.6	785.6	3	0.49	19.2
89	393	0	192	0	940.6	785.6	90	0.49	48.85
90	393	0	192	0	940.6	785.6	28	0.49	39.6
91	480	0	192	0	936.2	712.2	28	0.4	43.94
92	480	0	192	0	936.2	712.2	7	0.4	34.57
93	480	0	192	0	936.2	712.2	90	0.4	54.32
94	480	0	192	0	936.2	712.2	3	0.4	24.4
95	333	0	192	0	931.2	842.6	3	0.58	15.62
96	289	0	192	0	913.2	895.3	7	0.66	14.6
97	333	0	192	0	931.2	842.6	28	0.58	31.97
98	333	0	192	0	931.2	842.6	7	0.58	23.4
99	289	0	192	0	913.2	895.3	28	0.66	25.57
100	333	0	192	0	931.2	842.6	90	0.58	41.68
101	393	0	192	0	940.6	785.6	7	0.49	27.74
102	397	0	185.7	0	1040.6	734.3	28	0.47	33.08
103	382.5	0	185.7	0	1047.8	739.3	7	0.49	24.07
104	295.8	0	185.7	0	1091.4	769.3	7	0.63	14.84
105	397	0	185.7	0	1040.6	734.3	7	0.47	25.45
106	381.4	0	185.7	0	1104.6	784.3	28	0.49	22.49
107	295.8	0	185.7	0	1091.4	769.3	28	0.63	25.22
108	339.2	0	185.7	0	1069.2	754.3	7	0.55	21.18
109	381.4	0	185.7	0	1104.6	784.3	7	0.49	14.54
110	339.2	0	185.7	0	1069.2	754.3	28	0.55	31.9
111	382.5	0	185.7	0	1047.8	739.3	28	0.49	37.44
112	339	0	197	0	968	781	3	0.58	13.22
113	339	0	197	0	968	781	7	0.58	20.97
114	339	0	197	0	968	781	14	0.58	27.04
115	339	0	197	0	968	781	28	0.58	32.04
116	339	0	197	0	968	781	90	0.58	35.17
117	339	0	197	0	968	781	180	0.58	36.45
118	339	0	197	0	968	781	365	0.58	38.89
119	277	0	191	0	968	856	14	0.69	21.26
120	277	0	191	0	968	856	28	0.69	25.97
121	277	0	191	0	968	856	3	0.69	11.36
122	277	0	191	0	968	856	90	0.69	31.25
123	277	0	191	0	968	856	180	0.69	32.33
124	277	0	191	0	968	856	360	0.69	33.7
125	307	0	193	0	968	812	180	0.63	34.49
126	307	0	193	0	968	812	365	0.63	36.15
127	307	0	193	0	968	812	3	0.63	12.54
128	307	0	193	0	968	812	28	0.63	27.53
129	307	0	193	0	968	812	90	0.63	32.92
130	325	0	184	0	1063	783	7	0.57	17.54
131	325	0	184	0	1063	783	28	0.57	30.57
132	275	0	183	0	1088	808	7	0.67	14.2
133	275	0	183	0	1088	808	28	0.67	24.5
134	300	0	184	0	1075	795	7	0.61	15.58
135	300	0	184	0	1075	795	28	0.61	26.85
136	375	0	186	0	1038	758	7	0.5	26.06
137	375	0	186	0	1038	758	28	0.5	38.21
138	400	0	187	0	1025	745	28	0.47	43.7
139	400	0	187	0	1025	745	7	0.47	30.14
140	350	0	186	0	1050	770	7	0.53	20.28
141	350	0	186	0	1050	770	28	0.53	34.29
142	310	0	192	0	1012	830	3	0.62	11.85
143	310	0	192	0	1012	830	7	0.62	17.24
144	310	0	192	0	1012	830	28	0.62	27.83
145	310	0	192	0	1012	830	90	0.62	35.76
146	310	0	192	0	1012	830	120	0.62	38.7
147	331	0	192	0	1025	821	3	0.58	14.31
148	331	0	192	0	1025	821	7	0.58	17.44
149	331	0	192	0	1025	821	28	0.58	31.74
150	331	0	192	0	1025	821	90	0.58	37.91
151	331	0	192	0	1025	821	120	0.58	39.38
152	349	0	192	0	1056	809	3	0.55	15.87
153	349	0	192	0	1056	809	7	0.55	9.01
154	349	0	192	0	1056	809	28	0.55	33.61
155	349	0	192	0	1056	809	90	0.55	40.66
156	349	0	192	0	1056	809	120	0.55	40.86
157	296	0	186	0	1090	769	7	0.63	18.91
158	296	0	186	0	1090	769	28	0.63	25.18
159	297	0	186	0	1040	734	7	0.63	30.96
160	480	0	192	0	936	721	28	0.4	43.89
161	480	0	192	0	936	721	90	0.4	54.28
162	397	0	186	0	1040	734	28	0.47	36.94
163	281	0	186	0	1104	774	7	0.66	14.5
164	281	0	185	0	1104	774	28	0.66	22.44
165	500	0	200	0	1125	613	1	0.4	12.64
166	500	0	200	0	1125	613	3	0.4	26.06
167	500	0	200	0	1125	613	7	0.4	33.21
168	500	0	200	0	1125	613	14	0.4	36.94
169	500	0	200	0	1125	613	28	0.4	44.09
170	540	0	173	0	1125	613	7	0.32	52.61
171	540	0	173	0	1125	613	14	0.32	59.76
172	540	0	173	0	1125	613	28	0.32	67.31
173	540	0	173	0	1125	613	90	0.32	69.66
174	540	0	173	0	1125	613	180	0.32	71.62
175	540	0	173	0	1125	613	270	0.32	74.17
176	350	0	203	0	974	775	7	0.58	18.13
177	350	0	203	0	974	775	14	0.58	22.53
178	350	0	203	0	974	775	28	0.58	27.34
179	350	0	203	0	974	775	56	0.58	29.98
180	350	0	203	0	974	775	90	0.58	31.35
181	350	0	203	0	974	775	180	0.58	32.72
182	385	0	186	0	966	763	1	0.48	6.27
183	385	0	186	0	966	763	3	0.48	14.7
184	385	0	186	0	966	763	7	0.48	23.22
185	385	0	186	0	966	763	14	0.48	27.92
186	385	0	186	0	966	763	28	0.48	31.35
187	331	0	192	0	978	825	180	0.58	39
188	331	0	192	0	978	825	360	0.58	41.24
189	349	0	192	0	1047	806	3	0.55	14.99
190	331	0	192	0	978	825	3	0.58	13.52
191	382	0	186	0	1047	739	7	0.49	24
192	382	0	186	0	1047	739	28	0.49	37.42
193	382	0	186	0	1111	784	7	0.49	11.47
194	281	0	186	0	1104	774	28	0.66	22.44
195	339	0	185	0	1069	754	7	0.55	21.16
196	339	0	185	0	1069	754	28	0.55	31.84
197	295	0	185	0	1069	769	7	0.63	14.8
198	295	0	185	0	1069	769	28	0.63	25.18
199	296	0	192	0	1085	765	7	0.65	14.2
200	296	0	192	0	1085	765	28	0.65	21.65
201	296	0	192	0	1085	765	90	0.65	29.39
202	331	0	192	0	879	825	3	0.58	13.52
203	331	0	192	0	978	825	7	0.58	16.26
204	331	0	192	0	978	825	28	0.58	31.45
205	331	0	192	0	978	825	90	0.58	37.23
206	349	0	192	0	1047	806	7	0.55	18.13
207	349	0	192	0	1047	806	28	0.55	32.72
208	349	0	192	0	1047	806	90	0.55	39.49
209	349	0	192	0	1047	806	180	0.55	41.05
210	349	0	192	0	1047	806	360	0.55	42.13
211	302	0	203	0	974	817	14	0.67	18.13
212	302	0	203	0	974	817	180	0.67	26.74
213	525	0	189	0	1125	613	180	0.36	61.92
214	500	0	200	0	1125	613	90	0.4	47.22
215	500	0	200	0	1125	613	180	0.4	51.04
216	500	0	200	0	1125	613	270	0.4	55.16
217	540	0	173	0	1125	613	3	0.32	41.64
218	339	0	185	0	1060	754	28	0.55	31.65
219	393	0	192	0	940	758	3	0.49	19.11
220	393	0	192	0	940	758	28	0.49	39.58
221	393	0	192	0	940	758	90	0.49	48.79
222	382	0	185	0	1047	739	7	0.48	24
223	382	0	185	0	1047	739	28	0.48	37.42
224	310	0	192	0	970	850	7	0.62	14.99
225	310	0	192	0	970	850	28	0.62	27.92
226	310	0	192	0	970	850	90	0.62	34.68
227	310	0	192	0	970	850	180	0.62	37.33
228	310	0	192	0	970	850	360	0.62	38.11
229	525	0	189	0	1125	613	3	0.36	33.8
230	525	0	189	0	1125	613	7	0.36	42.42
231	525	0	189	0	1125	613	14	0.36	48.4
232	525	0	189	0	1125	613	28	0.36	55.94
233	525	0	189	0	1125	613	90	0.36	58.78
234	525	0	189	0	1125	613	270	0.36	67.11
235	322	0	203	0	974	800	14	0.63	20.77
236	322	0	203	0	974	800	28	0.63	25.18
237	322	0	203	0	974	800	180	0.63	29.59
238	302	0	203	0	974	817	28	0.67	21.75
239	397	0	185	0	1040	734	28	0.47	39.09
240	480	0	192	0	936	721	3	0.4	24.39
241	522	0	146	0	896	896	7	0.28	50.51
242	522	0	146	0	896	896	28	0.28	74.99
243	374	0	190	7	1013	730	28	0.51	39.05
244	305	100	196	10	959	705	28	0.64	30.12
245	298	107	186	6	879	815	28	0.62	42.64
246	318	126	210	6	861	737	28	0.66	40.06
247	356	142	193	11	801	778	28	0.54	40.87
248	314	113	170	10	925	783	28	0.54	38.46
249	321	128	182	11	870	780	28	0.57	37.26
250	298	107	210	11	880	744	28	0.7	31.87
251	322	116	196	10	818	813	28	0.61	31.18
252	313	113	178	8	1002	689	28	0.57	36.8
253	326	138	199	11	801	792	28	0.61	40.68
254	336	0	182	3	986	817	28	0.54	44.86
255	298	107	164	13	953	784	28	0.55	35.86
256	313	0	178	8	1000	822	28	0.57	25.1
257	313.3	113	178.5	8	1001.9	688.7	28	0.57	36.8
258	326.5	137.9	199	10.8	801.1	792.5	28	0.61	38.63
259	336.5	0	181.9	3.4	985.8	816.8	28	0.54	44.87
260	298.1	107.5	163.6	12.8	953.2	784	28	0.55	35.87
261	312.7	0	178.1	8	999.7	822.2	28	0.57	25.1
262	374.3	0	190.2	6.7	1013.2	730.4	28	0.51	39.06
263	304.8	99.6	196	9.8	959.4	705.2	28	0.64	30.12
264	298.1	107	186.4	6.1	879	815.2	28	0.63	42.64
265	317.9	126.5	209.7	5.7	860.5	736.6	28	0.66	40.06
266	355.9	141.6	193.3	11	801.4	778.4	28	0.54	40.87
267	313.8	112.6	169.9	10.1	925.3	782.9	28	0.54	38.46
268	321.4	127.9	182.5	11.5	870.1	779.7	28	0.57	37.27
269	298.2	107	209.7	11.1	879.6	744.2	28	0.7	31.88
270	322.2	115.6	196	10.4	817.9	813.4	28	0.61	31.18
